# Uptake and speciation of zinc in edible plants grown in smelter contaminated soils

**DOI:** 10.1371/journal.pone.0226180

**Published:** 2020-04-17

**Authors:** Bhoopesh Mishra, Louis M. McDonald, Mimi Roy, Antonio Lanzirotti, Satish C. B. Myneni

**Affiliations:** 1 School of Chemical and Process Engineering, University of Leeds, Leeds, England, United Kingdom; 2 Department of Physics, Illinois Institute of Technology, Chicago, Illinois, United States of America; 3 Division of Plant and Soil Sciences, West Virginia University, Morgantown, West Virginia, United States of America; 4 Jindal School of Liberal Arts and Humanities, OP Jindal Global University, Sonipat, Haryana, India; 5 GeoSoil CARS, University of Chicago, Chicago, Illinois, United States of America; 6 Department of Geosciences, Princeton University, Princeton, New Jersey, United States of America; Guangdong Technion Israel Institute of Technology, CHINA

## Abstract

Heavy metal accumulation in edible plants grown in contaminated soils poses a major environmental risk to humans and grazing animals. This study focused on the concentration and speciation of Zn in different edible plants grown in soils contaminated with smelter wastes (Spelter, WV, USA) containing high levels of the metals Zn, Cu, Pb, Cd. Their accumulation was examined in different parts (roots, stem, and leaves) of plants and as a function of growth stage (dry seed, sprouting seed, cotyledon, and leaves) in the root vegetables radish, the leafy vegetable spinach and the legume clover. Although the accumulation of metals varied significantly with plant species, the average metal concentrations were [Zn] > [Pb] > [Cu] > [Cd]. Metal uptake studies were complemented with bulk and micro X-ray absorption spectroscopy (XAS) at Zn K-edge and micro X-ray fluorescence (μXRF) measurements to evaluate the speciation and distribution of Zn in these plant species. Dynamic interplay between the histidine and malate complexation of Zn was observed in all plant species. XRF mapping of spinach leaves at micron spatial resolution demonstrated the accumulation of Zn in vacuoles and leaf tips. Radish root showed accumulation of Zn in root hairs, likely as ZnS nanoparticles. At locations of high Zn concentration in spinach leaves, μXANES suggests Zn complexation with histidine, as opposed to malate in the bulk leaf. These findings shed new light on the dynamic nature of Zn speciation in plants.

## Introduction

Soils in many parts of the world are contaminated by toxic metals and metalloids. Their sources may be linked to mining and ore smelting processes [1,2], solid and liquid wastes from municipal waste treatment plants [3], industrial activities [4], pesticides [5], fertilizers and liming materials [6], or the burning of fossil fuels [7]. Some metals, such as copper (Cu) and zinc (Zn), are essential for plants and animals at low concentrations but become toxic above a critical concentration. Other elements, such as cadmium (Cd), mercury (Hg) or lead (Pb), have no known function in plants and may already produce harmful effects at low concentrations [8]. These metals tend to persist in soil systems for a long time and potentially bioaccumulate in plants [9,10]. Feeding on edible plants grown in such contaminated soils can be an important exposure pathway for higher organisms, and thus poses a major environmental threat [11–13]. This study examined the uptake, distribution and speciation of Zn in spinach (*Spinacia oleracea*), radish (*Raphinus sativus*) and clover (*Trifolium repens*), which were grown in soils contaminated with smelter waste materials. The main focus was on radish and spinach, where the distribution of Zn was evaluated using μXRF imaging. In addition to the detailed investigation of Zn uptake, distribution and speciation, the bioaccumulation of Pb, Cd and Cu in plants was also determined.

Zinc is an essential element for plant metabolism and growth. It is a constituent of metalloenzymes, or a cofactor for several enzymes such as anhydrases, dehydrogenases, oxidases and peroxidases, and plays an important role in regulating nitrogen metabolism, cell multiplication, photosynthesis, and auxin synthesis in plants [14,15]. It also plays an important role in the synthesis of nucleic acids and proteins and helps in the utilization of phosphorous and nitrogen during seed formation. While Zn is crucial for the above-mentioned processes, high levels of uncomplexed Zn are toxic to plants, and Zn is associated with the blockage of xylem elements and the inhibition of photosynthesis through alteration of electron transport and the capacity of rubisco to fix CO_2_ [16]. Common Zn toxicity symptoms in plants are stunted growth of shoots, curling and rolling of young leaves, death of leaf tips, and chlorosis.

To deal with toxic levels of Zn, plants possess a range of potential detoxification mechanisms for Zn homeostasis that allow uptake and distribution of Zn to tissues while maintaining Zn within cells or subcellular compartments below toxic levels [17–19]. Tolerance mechanisms for Zn could include restricted uptake, chelation by organic acids and polypeptides, and isolation in vacuoles [20,21]. Previous studies have shown that Zn accumulates in vacuoles of plants [22–24]. In addition to accumulation in vacuoles, complexation of metals by carboxylic and amino acids (e.g. malate, histidine) has been suggested to play an important role in tolerance and detoxification of heavy metals [17, 25]. Because of the high affinity of Zn with S, N, and O, carboxylic and amino acids are potential ligands for Zn complexation and their availability can aid detoxification [25]. Malate, a carboxylic group ligand with high binding affinity for Zn, was proposed as a cytosolic Zn chelator in Zn tolerant plants [26]. However, unequivocal evidence for overexpression of specific organic acids to counter Zn toxicity in plants has not been established.

In the last two decades, a number of X-ray Absorption Spectroscopy (XAS) studies have focused on the speciation of Zn in hyperaccumulator plants. In *Thlaspi caerulescens*, Zn was reported to be present predominantly in Zn-histidine complexes in mature leaves and roots and stems [27, 28], but tended to coordinate with O-ligands, likely Zn-malate or–citrate, under conditions of Cd toxicity [28]. In *Arabidopsis halleri*, octahedrally coordinated malate complexes were reported to be predominant under a variety of conditions, but roots of *A*. *halleri* as well as roots and leaves of the related *A*. *lyrata* showed predominant Zn-phytate complexes [29]. Kelly et al. [30] found that in *Datura innoxia*, Zn coordination with mixed N/O ligation varied from 4-coordinate to 5 with increasing Zn^2+^ solution concentrations (250–500 mM to 1000 mM). Most recently, a number of studies on the hyperaccumulator *Brassica juncea* looked at Zn speciation in the plant roots in the presence and absence of different plant growth promoting bacteria [31], different contaminant species such as ZnSO_4_, and nanoparticles of ZnO and ZnS [32]. They concluded that Zn speciation in the roots depended strongly on all of the studied factors. While plants grown in ZnSO_4_ and sterile soil predominantly contained Zn phytate (35%) and polygalacturonate (30%), the presence of bacteria shifted speciation more towards Zn cysteine and, in the case of ZnO nanoparticle contamination, to Zn histidine [32].

There are only few studies on speciation of Zn in a non-accumulating plant [33, 34]. Terzano et al. conducted a study on *Eruca vesicaria* L. *Cavalieri*, a common edible plant in the Mediterranean, in Zn-contaminated soil, and compared Zn speciation in the plants after different soil amendments were applied [33]. While total Zn concentration remained similar, they found that plants grown in contaminated soils contained Zn-phosphates and oxalates, plants grown on compost-amended soil complex Zn as phytate, and citrate in roots, and as phosphate, cysteine and histidine in leaf cells, but also succeeded in compartmentalizing the Zn better. Sarret et al. investigated the relationship between the chemical form and localization of Zn in progenies *Arabidopsis halleri* and *Arabidopsis lyrate* plant leaves and their Zn accumulation capacity [34]. Elemental mapping of the leaves revealed different Zn partitioning between the veins and the leaf tissue. The study found Zn bound predominantly with O groups, either complexed to a combination of organic acids or as aqueous Zn. Their results support the role of carboxyl and/or hydroxyl groups as major Zn ligands in trichomes.

These studies demonstrate the variability of Zn complexation in plants, mostly focusing on hyperaccumulators. Spectroscopic evidences of histidine, malate, citrate, phytate, phosphate and cysteine show their important role in Zn sequestration and compartmentalization in plant species, suggesting different plants may utilize different Zn sequestration strategies. Studies conducted on hyperaccumulators may not be applicable for Zn speciation in other types of plants under elevated Zn conditions.

The objective of this study is to determine Zn uptake and understand the speciation and distribution of Zn in vegetables grown in soils contaminated by Zn smelter wastes. Since Zn smelter waste contaminated soil was co-contaminated with Cd, Cu, and Pb, co-uptake of these metals was also measured. However, speciation and distribution studies of this work focused on Zn in the root vegetable radish, the leafy spinach, as well as clover, using XAS. To complement the bulk XAS study, X-ray spectromicroscopy (μXRF) was used to understand the μm-scale spatial distribution and speciation of Zn in radish root and spinach leaves.

## Materials and methods

### Soil sampling and characterization

**Fig 1:**
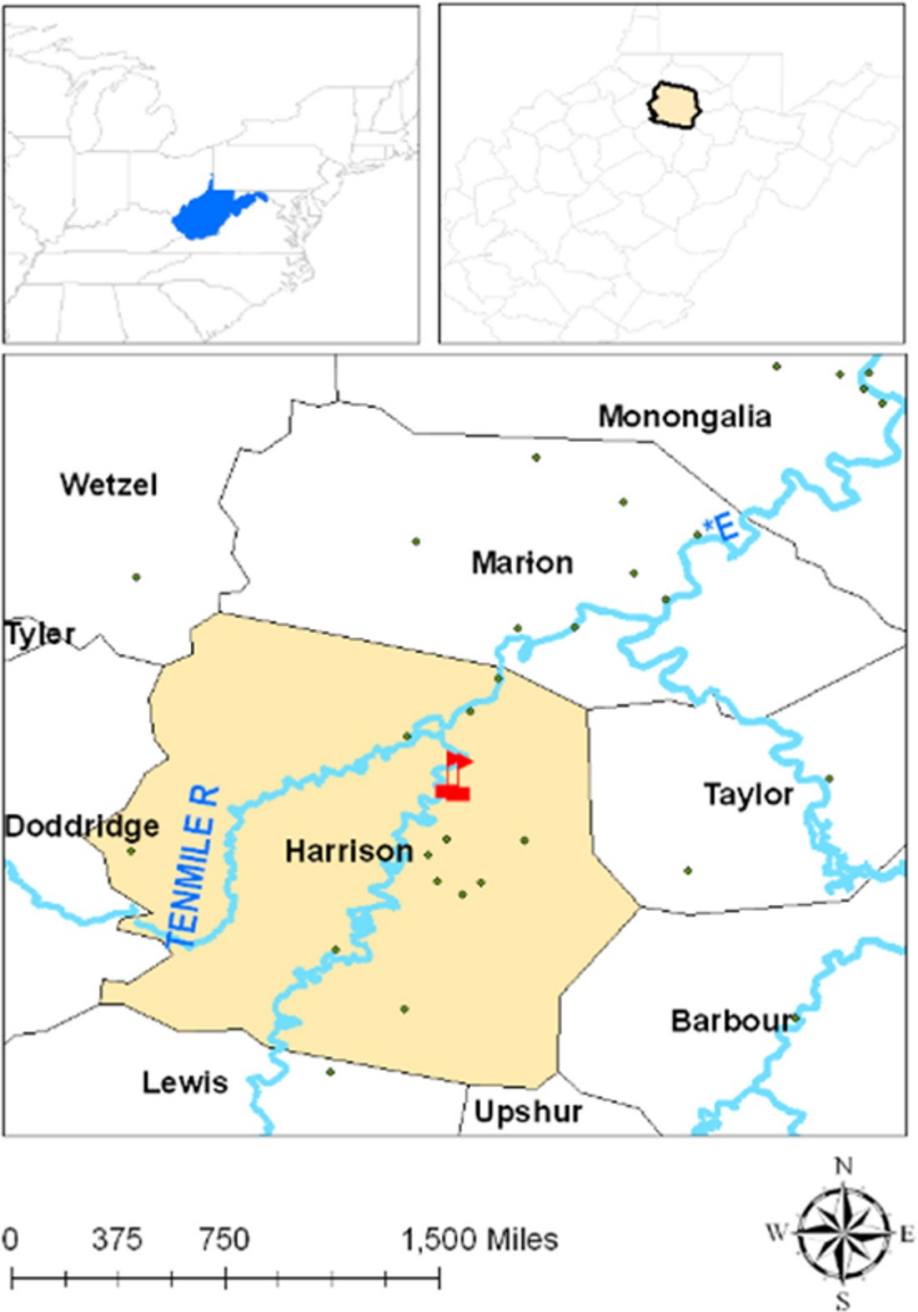
Location and coordinates of soil sampling site in West Virginia, USA.

Spelter (WV, USA) is the site of a former Zn smelting plant (Fig 1). The approximate coordinates for the location are 39° 20’ 42” N and 80° 19’ 04” W. Improper disposal of wastes and byproducts resulted in contamination of an area of approx. 20 ha, ranging in depth from a few centimeters at the margins to 30 m at the deepest point. Located adjacent to the West Fork River, waste disposal has contaminated the river and its sediments as well as soils in the residential areas, including a playground. Reports of contamination at this site has been published in Roy and McDonald [36]. Contaminated soil was collected across the river from a terrace less than 200 m from the edge of the disposed waste that had been mapped as Urban Land, but based on its location and nearby mapped soils was likely a Lindside (Fine-silty, mixed, active, mesic Fluvaquentic Eutrudepts). Approximately 480 kg of soil were collected for a larger study on smelter soil. Permits were not required for the access of the site because this site is not a controlled or regulated site. The soil was air-dried, crushed to pass a 2 mm sieve, and stored in plastic tubs.

The pH of soil suspension (prepared using 1:1 distilled deionized water and soil) was measured with a glass membrane electrode, texture by the pipette method, effective cation exchange capacity (ECEC) by the sum of NH_4_Cl extractable Al, Ca, Mg, Na, and K (Sumner and Miller, 1996), and soil carbon by dry combustion (TruSpec CHN analyzer, LECO Corp. St. Joseph, MO). To determine the ‘total’ Zn, Cu, Cd and Pb contents of the soil, a microwave-assisted (MARS 5, CEM Corp., Matthews, NC) digestion in trace-metal grade HNO_3_ was conducted. On separate soil aliquots, operationally defined ‘soluble’ and ‘exchangeable’ metal fractions were determined using sequential extraction. For the ‘soluble’ fraction, metal concentrations were determined in the deionized water extracts from soil suspensions (2.0 g soil in 16.0 mL). The ‘exchangeable’ fraction was determined after Fraction 1 of the Tessier et al. (1979) method by subsequently extracting the 2.0 g soil residue using 16.0 mL 1.0 M MgCl_2_. [35] All metal concentrations were determined by ICP-OES (Perkin-Elmer Optima 2100DV, Norwalk, CT). Further reference to the methods can be found in Roy and McDonald [36].

### Plant growth and uptake experiments

The plants were grown in a greenhouse setting. Day and night temperatures, relative humidity, and pan evaporation rate were not recorded since the study focused on growth trials under similar growth conditions using contaminated soil obtained from field setting. Water was added through sprinklers in the morning and evening after sowing. After germination, the pots were removed from the seed bed and watered manually on a regular basis. The pots were drained throughout the course of the experiment. The pots were grown under natural daylight and in artificial light.

Seeds of radish (*Raphinus sativus*), spinach (*Spinacia oleracea*), and clover (*Trifolium repens*) were sown into separate pots (five replicates of each) of moistened contaminated Spelter soil, placed in a mist bed and brought to approximately pot capacity. After germination, pots were removed from the mist bed and watered by hand as needed. At maturity, root, shoot and leaf tissues were harvested separately and rinsed of adhering soil by sonication. Then they were dried (90°C for 72 h), ground, microwave digested, and Cd, Cu, Pb and Zn concentrations were determined as described above. Spinach and radish seeds were planted in the same way as described above, but different replicates were harvested at different times: as dry seeds, seeds that had imbibed soil solution from the Spelter soil, cotyledons after 10 days of sowing, and leaves after 90 days of sowing. They were dried, ground, microwave digested in trace metal grade HNO_3_ and analyzed as described above. All metal concentrations were converted to an oven-dry basis. Data were analyzed using the statistical analysis software SAS [37] by considering species and plant part (stem, leaf, root or dry seed, sprouting seed, cotyledon) as categorical variables in the generalized linear model (GLM) procedure. Means were separated by least significant difference (α = 0.05). The bioconcentration factor (BCF) was calculated as the ratio of metal concentration on the plant tissue over the initial concentration of the metal in the soil in, as well as over the initial soluble concentration of the metal [38]

Samples comprising of 10–12 seeds or plants were wrapped in moist tissue, placed in plastic petri dishes, sealed with Parafilm and shipped overnight to the National Synchrotron Light Source (NSLS) for XAS analysis.

### XAFS (XANES and EXAFS) measurements and data reduction

#### XAFS measurements and data reduction

X-ray Absorption Fine Structure (XAFS) measurements were performed at beamline X18B of NSLS on the Zn K edge, collecting both X-ray Absorption Near Edge Structure (XANES) and Extended X-ray Absorption Fine Structure (EXAFS) spectra. The energy of the incident X-rays was scanned using a Si(111) reflection plane of a cryogenically-cooled double-crystal monochromator. Since X18B is a bending magnet beamline, variation in the incident photon intensity for the scanned energy range was less than 15%. Higher harmonics were removed by detuning the second monochromator crystal by 30%. Samples with higher Zn concentration (powdered standards) were measured in transmission mode while dilute samples (plant samples and aqueous standards) were measured in fluorescence mode. The ionization chamber used to measure the incident photons was filled with a mixture of 50% N_2_and 50% He, while the ion chambers used to measure the sample transmission were filled with a mixture of 60% N_2_ and 40% Ar gas. An energy dispersive solid state (13-element) Canberra Ge detector was used for the fluorescence mode detection of the XAFS signal.

Samples were loaded into a slotted Plexiglas holder and covered with Kapton film for XANES and EXAFS measurements. For bulk XAFS analysis, plant parts were ground before mounting into the sample holder. Energy scans of each sample were collected with a step size of 0.5 eV near the edge, 5.0 eV below the edge, and 0.07 *k* (Å^-1^) above the edge with a dwell time of 5–15 seconds per point depending on the signal to noise ratio of the sample. Consecutive spectra were monitored for possible radiation-induced changes. A total of 5 to 10 consecutive scans were collected for each sample depending on the Zn concentration in the sample. All scans were calibrated using a simultaneously collected spectrum of a Zn foil (first inflection point in the spectrum at 9659 eV), and averaged before further data analysis.

The XAFS spectra were analyzed using the UWXAFS package [39]. Processing of the raw data, including the alignment of spectra and background removal, were implemented using ATHENA [40]. The maximum frequency of the background was set to 1.0 Å for background subtraction. The χ(k) data range used for EXAFS Fourier transforms was 2.3–9.5 Å^-1^. A Hanning window function with a dk value of 1.0 Å^-1^ was used to avoid truncation ripples in the Fourier transform [41]. Multiple k-weight (*k*^1^, *k*^2^, *k*^3^) fitting of each spectrum was followed by simultaneous data fits. The fitting range for all of the Fourier transforms was 1.2–3.8 Å for standards and 1.1–2.6 Å for plant samples.

### XAFS standards

Powder and aqueous Zn standards were used to provide references for Zn binding environments with various ligands. Zn–oxide, acetate, phosphate, sulfate, sulfide, and carbonate powder standards were prepared by grinding (passing through ~ 400 mesh) the commercially available chemicals (Sigma-Aldrich). The aqueous Zn standards were aqueous Zn^2+^, Zn(ClO_4_)_2_, Zn-malate, Zn-cysteine, Zn-citrate, and Zn-histidine solutions. All the aqueous Zn standards were prepared at pH 7.0 using 10 mM Zn in a 1:10 Zn:ligand ratio. Further, Visual Minteq was used to calculate the solution speciation of all the aqueous standards under the experimental conditions described above [42]. At Zn:ligand ratio of 1:10 and pH 7, chemical speciation calculation using Visual Minteq suggests the formation of Zn-malate and Zn-(histidine)_2_, and Zn-(cysteine)_4_ as the dominant species (>85%) using 5 mM Zn^2+^ in solutions.

EXAFS data analysis presented here is based on refining theoretical EXAFS spectra (calculated using FEFF8) against the experimental data. The crystallographic information of the well characterized standard compounds (the powder standards) was used to generate a cluster of atoms (using ATOMS; [43]), and to generate theoretical EXAFS spectra (using FEFF8; [44]). Further, FEFFIT [45] was used to fit the EXAFS spectra of powdered Zn standards. Spectral fitting of the powder standards of Zn–oxide, acetate, phosphate, sulfate, sulfide, and carbonate was done from 1 to 4 Å, and was found to be in good agreement with the theoretical spectra generated by FEFF. The scattering paths used for fitting powder standards provided signatures for O/N, C, P, and S signals. Scattering paths representing O/N, C, P, and S signals were used for fitting the aqueous solution standards. Best fit values obtained from fitting aqueous solution standards were used as the initial guess parameters for unknown plant samples.

The value obtained for the EXAFS amplitude reduction factor (S0^2^) for all standards was 1.00 ± 0.05, and this value was used in modeling the data from the plant samples. Statistically significant lower R factor and *χ*_*v*_^*2*^ values were used as criteria for improvement in the fit to justify the addition of an atomic shell to the model [46].

### X-ray fluorescence mapping

Micro-X-ray fluorescence (μXRF) mapping of Zn in plant samples was conducted at beamline X26A of the NSLS. An energy dispersive Canberra 9-element Ge array detector was used for μXRF mapping using 5x10μm beam. The intact plant samples were glued to slotted paper cardboards and scanned with a step size of either 10 or 30 μm and 2 seconds per pixel counting time. Plant samples were exposed directly to the beam. μXRF mapping of spinach leaves and radish roots was conducted to examine the distribution of Zn. μXRF maps were followed by the micro-XANES (μXANES) measurements in regions of high Zn concentration (further referred to as ‘hotspots’).

## Results

### Soil characterization

The soil texture was a silt loam with a pH of 5.8, typical of soils in West Virginia. The relatively low cation exchange capacity (CEC) of 2.8 cmol_c_ kg^-1^ likely reflects that exchangeable contaminant ions were not accounted for in the CEC procedure. The organic carbon content was 5.8%, which is likely overestimated due to the black carbonaceous byproduct in the smelter waste material and should not be considered representative of soil organic matter. All examined total metal concentrations were above what has been reported for uncontaminated soils in the state [47]; Cu concentration was approximately five times higher; Cd and Pb 30 to 40 times; and Zn over 100 times higher (Table 1). Of the total soil concentrations, bioavailable plant forms (soluble + exchangeable) were about 4.4% for Zn, 3% for Cd, 0.4% for Cu and 1.2% for Pb (Table 2). Nonetheless contaminated soil resulted in significantly lower yield of plant biomass compared to the plants grown in control soil (Table 3). From extraction results, it is expected that the Zn in soil would be partitioned in several species.

**Table 1:** Properties of soils used.

Variables	Contaminated	Control
pH	4.45	6.5
Organic Carbon (%)	5.76	1.4
CEC (meq/100g)	0.9	3
Zn (mg/kg)	8600	70.6
Pb (mg/kg)	850	11.17
Cd (mg/kg)	42	1.83
Cu (mg/kg)	403	38.07
Fe (mg/kg)	16700	25557
Mg (mg/kg)	1182	988
Mn (mg/kg)	182	1250

**Table 2:** Calculation of bioconcentration factors for all measured plant parts and all measured metals.

	Bioconcentration Factor (BCF) = metal conc. in plant tissue [mg/kg] / initial conc. of metal in soil [mg/kg]											
	Zn			Pb			Cu			Cd		
Vegetable	Zn	BCF total conc	BCF soluble	Pb	BCF—total conc	BCF soluble	Cu	BCF—total conc	BCF soluble	Cd	BCF—total conc	BCF soluble
Unit	mg kg^-1^	/		mg kg^-1^			mg kg^-1^			mg kg^-1^		
Total conc. in soil	8600			850			200.0			96		
Soluble conc. in soil	210			1.2			0.5			12		
Exchangeable conc. In soil	170			8.6			0.3			19		
Radish root	800	0.09	3.8	38	0.04	31.7	18	0.09	36.0	4	0.04	0.33
Radish stem	360	0.04	1.7	110	0.13	91.7	29	0.15	58.0	28	0.29	2.33
Radish leaf	590	0.07	2.8	141	0.17	117.5	13	0.07	26.0	1	0.01	0.08
Spinach root	260	0.03	1.2	10	0.01	8.3	7	0.04	14.0	1	0.01	0.08
Spinach stem	460	0.05	2.2	27	0.03	22.5	19	0.10	38.0	6	0.06	0.50
Spinach leaf	1400	0.16	6.7	29	0.03	24.2	49	0.25	98.0	20	0.21	1.67
Clover root	220	0.03	1.1	23	0.03	19.2	6	0.03	12.0	2	0.02	0.17
Clover stem	200	0.02	1.0	10	0.01	8.3	9	0.05	18.0	3	0.03	0.25
Clover leaf	135	0.02	0.6	20	0.02	16.7	6	0.03	12.0	6	0.06	0.50

**Table 3:** Biomass for growth trial in contaminated as well as control soil.

Contaminated soil	Root	Stem	Leaf	Control Soil	Root	Stem	Leaf
	in grams/plant				in grams/plant		
**Radish**	0.59	0.42	0.79	**Radish**	3.79	1.62	2.79
	0.48	0.35	0.75		3.88	1.48	2.65
	0.39	0.27	0.72		3.69	1.51	2.72
	0.4	0.29	0.82		3.45	1.53	2.64
	0.29	0.38	0.8		3.72	1.58	2.58
					3.706	1.544	2.676
**Spinach**	0.32	0.15	0.27	**Spinach**	1.58	1.15	4.24
	0.35	0.11	0.29		1.62	1.08	4.05
	0.24	0.09	0.22		1.49	1.17	4.16
	0.26	0.11	0.25		1.56	1.08	4.13
	0.21	0.1	0.27		1.51	1.1	4.09

### Metal uptake by plants

#### Zinc (Zn)

**Fig 2:**
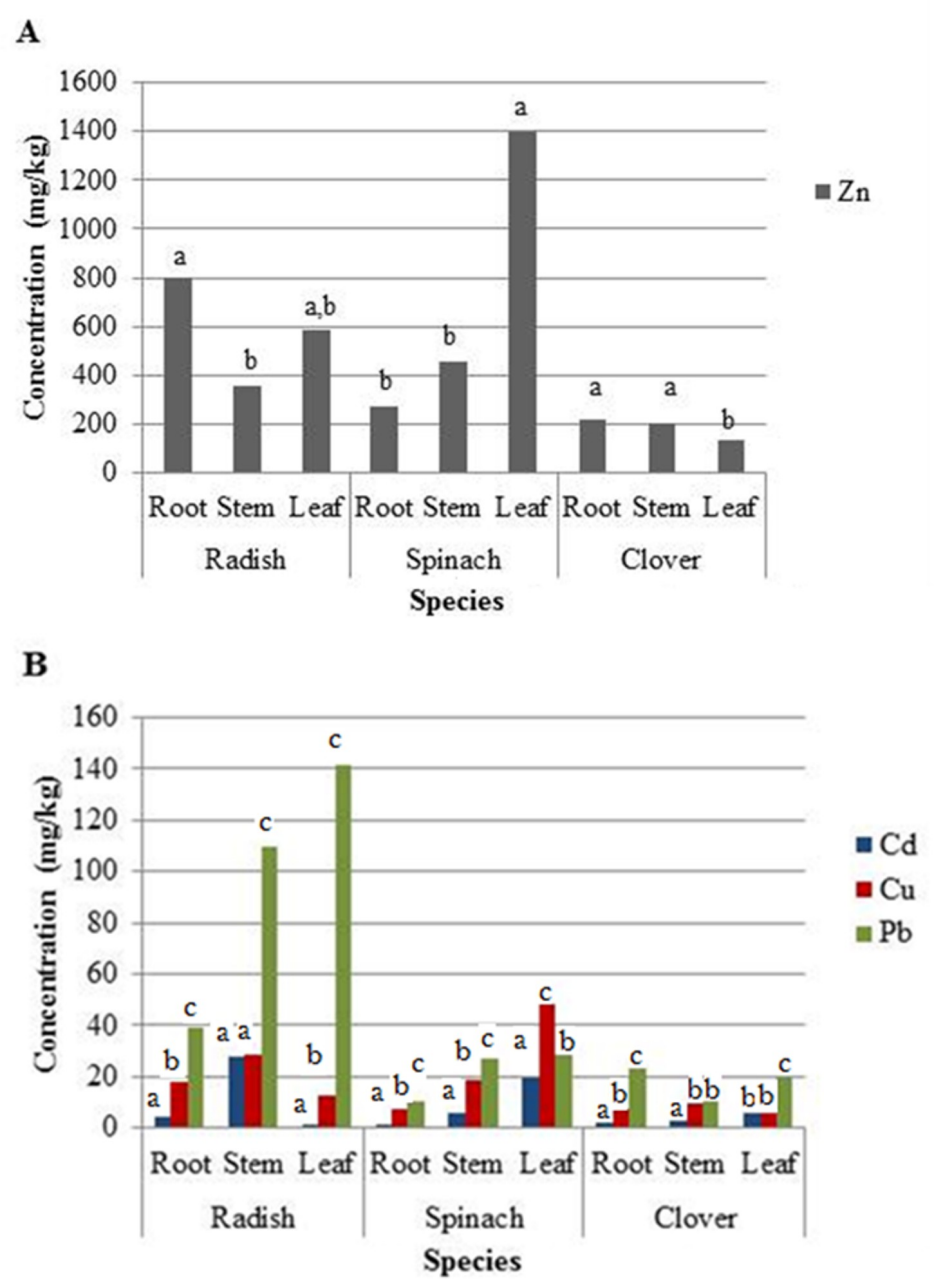
Concentrations of Zn (a) and Cd, Cu, and Pb (b) in the root, stem, and leaf of radish and spinach plants. Lower case letters indicate significant differences at 95% confidence.

**Fig 3:**
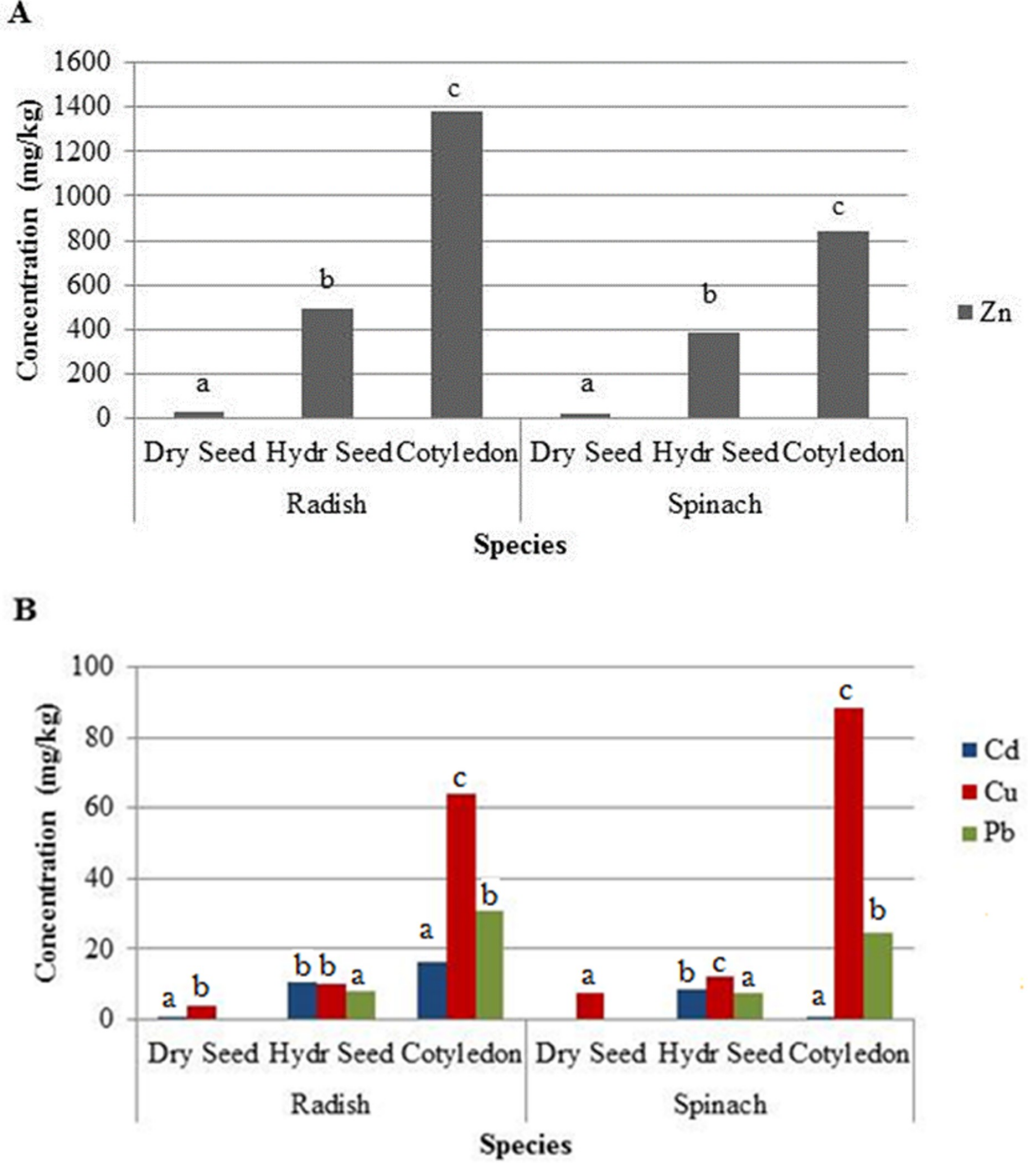
Concentrations of Zn (a) and Cd, Cu, and Pb(b) in the dry seed, sprouting seed, and cotyledon of radish and spinach. Lower case letters indicate significant differences at 95% confidence.

Zn uptake varied significantly between plant species (p < 0.0001), plant part (p = 0.0015) and a significant species-by-part interaction (p < 0.0001) on the uptake of Zn (R^2^ = 0.93). Zn concentrations in all plant parts resulted in bioconcentration factors (BCF) above 1 compared with the soluble soil Zn fraction, indicating plant Zn concentrations to be higher than soluble soil concentrations (Table 2). The largest Zn plant tissue concentration (1400 mg kg^-1^, BCF = 6.7) was found in spinach leaves (Fig 2A). Radish and spinach exhibited concentrations higher than the bioavailable soil fraction of 380 mg kg^-1^ in at least one plant part and Zn was > 200 mg kg^-1^ in all plant parts. In radish and clover, more zinc accumulated in roots and stems than in leaves (Fig 2A). Looking at Zn concentrations during the growth stages of radish and spinach, significant effects (α ≤ 0.05) could also be seen (Fig 3A). From dry seed to hydrated seed and cotyledon, there was a clear trend in both plant species for enhanced bioaccumulation in tissues as seeds imbibed water from the contaminated soil and cotyledons emerged and expanded.

#### Lead (Pb)

All measured Pb concentrations in plants were higher than expected based on the low ‘soluble’ soil concentrations, as shown by the high calculated BCFs. The single largest Pb concentration was found in radish, where leaves contained 140 mg kg^-1^ Pb (Fig 2B; Least Statistical Difference (LSD) = 14.3), even though extractions suggested that the soil only contained 9.8 mg kg^-1^ soluble Pb. Neither spinach nor clover accumulated more than 30 mg kg^-1^ in any plant part. The fact that radish and spinach seemed to translocate significant quantities of Pb to the above ground structures (stems and leaves) is surprising, considering a body of literature suggesting that Pb is generally relatively immobile, and is found primarily in roots of edible plants (e.g. [36, 48, 49]). Synergy in the co-uptake of Pb in the presence of high levels of Zn and other metals could possibly explain this observation. Differences in Pb concentration by plant part in clover were small and not statistically significant.

#### Cadmium (Cd)

All measured Cd plant concentrations were lower than the bioavailable soil fraction of 31 mg kg^-1^, as seen in the BCF values <1, except for spinach leaf and radish stem. The single highest concentration was found in radish stem (Fig 2B; LSD = 2.2). Spinach and clover showed a trend of increasing Cd from roots to the leaves, above-ground.

#### Copper (Cu)

The only essential element among the studied co-contaminants, Cu, had the lowest bioavailable soil fraction of 0.8 mg kg^-1^. There were significant differences in Cu concentration for each plant part for all plants except clover (Fig 2B; LSD = 2.9), with a tendency to increase from roots to leaves in spinach. Radish showed highest Cu concentrations in the stems. The highest absolute concentrations were measured in radish stems and spinach leaves. Radish was the only plant where the highest metal concentrations of Cd and Cu were found in the stems.

Examining the evolution of metal concentrations across different growth stages, much clearer trends could be established. As expected, Pb and Cd concentrations in dry radish and spinach seeds were negligible before planting (Fig 3B). Concentrations of Zn, Pb and Cu increased significantly as both spinach and radish seed imbibed contaminated soil solution and expanded to cotyledons. The largest change in tissue concentrations was between the sprouting seed and the expanded cotyledon. It appears that the cotyledons were able to bioaccumulate metals as they transpired. The lack of Casparian Strip [14] in cotyledon root, which affords mature roots some degree of metal accumulation selectivity, could explain the high bioaccumulation of metals in cotyledons.

### XAFS on Zn model compounds

Crystalline compounds of Zn-oxide, -phosphate, -acetate, -sulfate, -sulfide, and–carbonate were used to obtain unequivocal signatures of scattering paths of Zn-O, P, C, S, and Zn. Theoretically derived single scattering paths (using FEFF8 simulations of known crystal structure) were used to fit crystalline model compounds as well as for fitting the aqueous standards (Zn-malate, -cysteine, and–histidine complexes).

**Fig 4:**
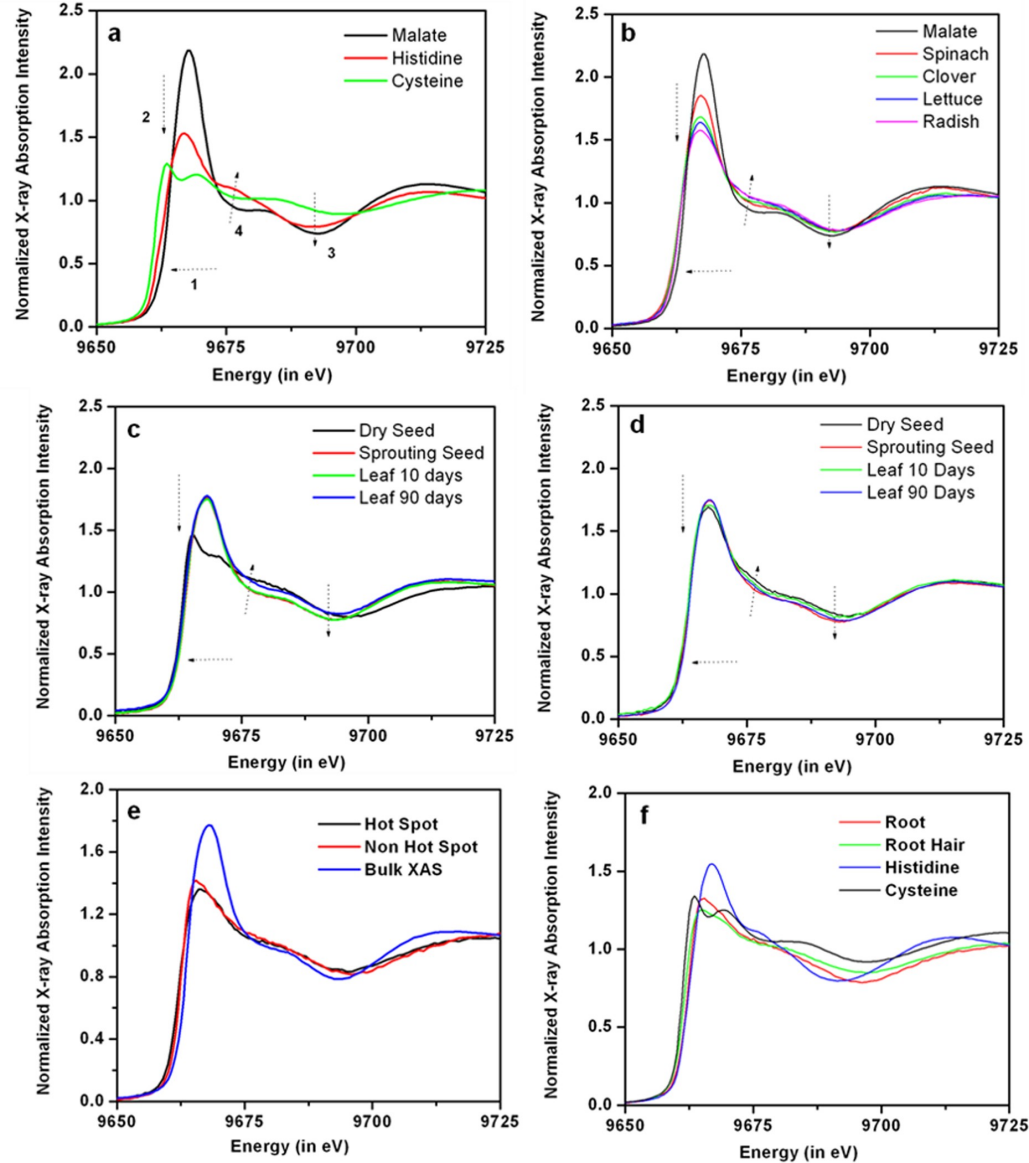
Normalized XANES data. (a) Zn solution standards, (b) spinach, radish, and clover cotyledons, (c) radish dry seed, sprouting seed, cotyledon, and leaf, (d) and spinach, (e) bulk, hot-spot, and non-hot spot of spinach leaf, (f), and main root and root hair of radish root.

Zn K-edge XANES of the aqueous complexes of Zn-histidine, -malate and -cysteine standards display significantly different spectral features (Fig 4A): energy of the absorption edges increase in the order cysteine < histidine < malate (arrow 1, Fig 4A); cysteine and histidine complexes of Zn exhibit much lower and broader white lines than malate complexes (arrow 2, Fig 4A); malate and histidine complexes exhibited oscillatory features at 9690 eV, which are missing for cysteine complexes (arrow 3, Fig 4A); histidine showed a more pronounced shoulder at about 9675 eV compared to malate (arrow 4, Fig 4A). In this study, these differences in the XANES spectra of Zn -malate, -cysteine, and -histidine complexes were used to distinguish them in unknown samples, providing a semi-quantitative fingerprint of their relative occurrence in each sample. These results provided the basis for more detailed analyses of the coordination environments of Zn using EXAFS fitting of solution standards (discussed below).

**Fig 5:**
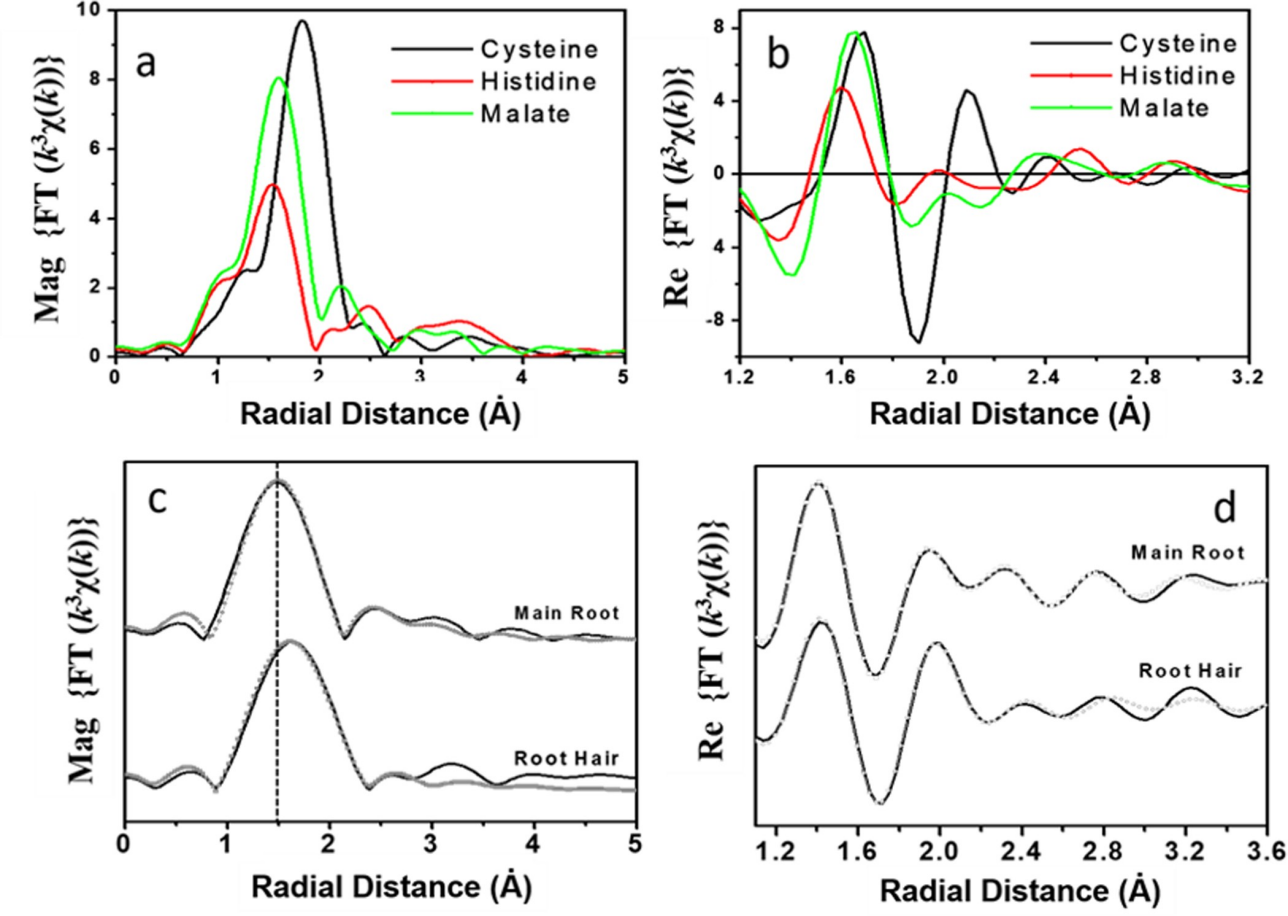
Fourier transform magnitudes of measured samples. (a) Zn solution standard data, (b) expanded real part of the Fourier Transform data of the Zn standard data, (c) Fourier Transform magnitude and fit of the radish root data collected on the main and root hair, (d), expanded real part of the Fourier Transform and fit of the radish root data collected on the main and root hair.

The EXAFS fitting results for the aqueous Zn standards are listed in Table 4. The magnitude of the Fourier transform of aqueous solution standards and the expanded real part of the FT are shown in Fig 5A and 5B. The cysteine standard displayed characteristic spectral features including an increase in the amplitude of the first peak and a shift of the first peak towards longer distance which are characteristic of backscattering from a heavier element (e.g., S compared with O/N) (Fig 5A and 5B). Although it is relatively easy to distinguish between O and S signals, it is often hard to distinguish between O and N signal using Zn EXAFS in a mixed O/N environment of an unknown sample due to the similar scattering amplitude and phase of O and N atoms. Differences between the Zn-histidine and Zn-malate spectra are discussed below.

**Table 4:** EXAFS fitting parameters for aqueous standards.

Sample	Path	CN	R (Ǻ)	σ^2^(Ǻ^2^)	ΔE_0_ (eV)
Malate	O	5.98 (±0.15)	2.08 (±0.02)	0.0101 (±0.0032)	4.6 (±1.4)
	C	2.85 (±0.45)	2.85 (±0.03)	0.0056 (±0.0022)	
Histidine	N	3.82 (±0.24)	2.03 (±0.02)	0.0067 (±0.0021)	3.1 (±0.6)
	O	1.25 (±0.36)	2.91 (±0.02)	0.0092 (±0.0034)	
	C	3.86 (±0.62)	3.01 (±0.03)	0.0051 (±0.0024)	
Cysteine	S	4.00*	2.29 (±0.02)	0.0081 (±0.0021)	4.8 (±1.9)

* Set to this value, based on published crystallographic structure.

At a Zn:ligand ratio of 1:10 and pH 7, chemical speciation calculations in Minteq suggest the formation of Zn-malate in malate-containing Zn solutions, and as Zn-(histidine)_2_ in histidine-containing Zn solutions. Zn is thus expected to be present as distorted octahedrally coordinated Zn-malate or tetrahedrally coordinated Zn-histidine complexes. The tetrahedral coordination geometry of Zn-histidine complex is reflected by a lower amplitude for the first peak of EXAFS Fourier transform (FT) data. The EXAFS FT for Zn malate, which has an octahedral coordination geometry, has a higher amplitude (Fig 5A and 5B). The smaller bond length for Zn-N in histidine complexes compared to Zn-O in malate complexes can be seen in the expanded real part of the Fourier transformed data (Fig 5B). In addition, histidine complexes can be identified by the strong multiple scattering feature arising from the imidazole ring around 3.8 Å (phase uncorrected). However, it is worth emphasizing here that the visual inspection of EXAFS data could be deceptive due to the interference of overlapping signals in a mixed coordination environment. To account for this, quantitative modeling of the aqueous solution standards (discussed below) was used to characterize the binding environments present in the plant samples. Finally, a combination of XANES and EXAFS data was used to provide an unambiguous interpretation of Zn -cysteine,–histidine, and–malate type binding environments in unknown plant samples.

The Zn-cysteine standard was fit with 4 S atoms in the first shell at a distance of 2.29 (± 0.02) Å, consistent with tetrahedral coordination environment. The coordination environment of Zn in the malate solution was modeled with O and C shells, corresponding to a complexed functional group. The Zn-malate standard was fit with 5.98 (± 0.15) O atoms in the first shell and 2.85 (± 0.45) C atoms in the second shell, indicating an octahedral coordination environment. The Zn-O and Zn-C distances in the Zn-malate standard was found to be 2.08 (± 0.02) Å and 2.85 (± 0.03) Å respectively, consistent with the published crystal structure [50]. The Zn histidine standard was fit with 3.82 (± 0.24) N atoms in the first shell and 3.86 (± 0.62) C atoms in the second shell, indicating a tetrahedral coordination environment. The Zn-N and Zn-C distance in the Zn-histidine standard was found to be 2.03 (± 0.02) Å, and 3.01 (± 0.03) Å respectively, also consistent with the published crystal structure [51]. The addition of ~ 1 O atom at 2.91(± 0.02) Å improved the quality of the fit. Further details about EXAFS data of the standards used here and their fit in k- as well as r- space has been presented elsewhere [52].

In summary, Zn-cysteine, -histidine, and -malate complexation could be identified using a combination of the features of XANES spectra and EXAFS bond distances. Zn-S bond distance is 2.29 (± 0.02) Å, much higher than Zn-O/N bond distances. The average Zn-O/N bond distances of the tetrahedral (e.g. histidine) and octahedral (e.g. malate) coordination environment of Zn species are in the range of 1.96 to 2.03 Å and 2.06 to 2.12 Å respectively.

### XAFS on plant samples

#### Zn speciation in cotyledons of radish, spinach and clover

**Fig 6:**
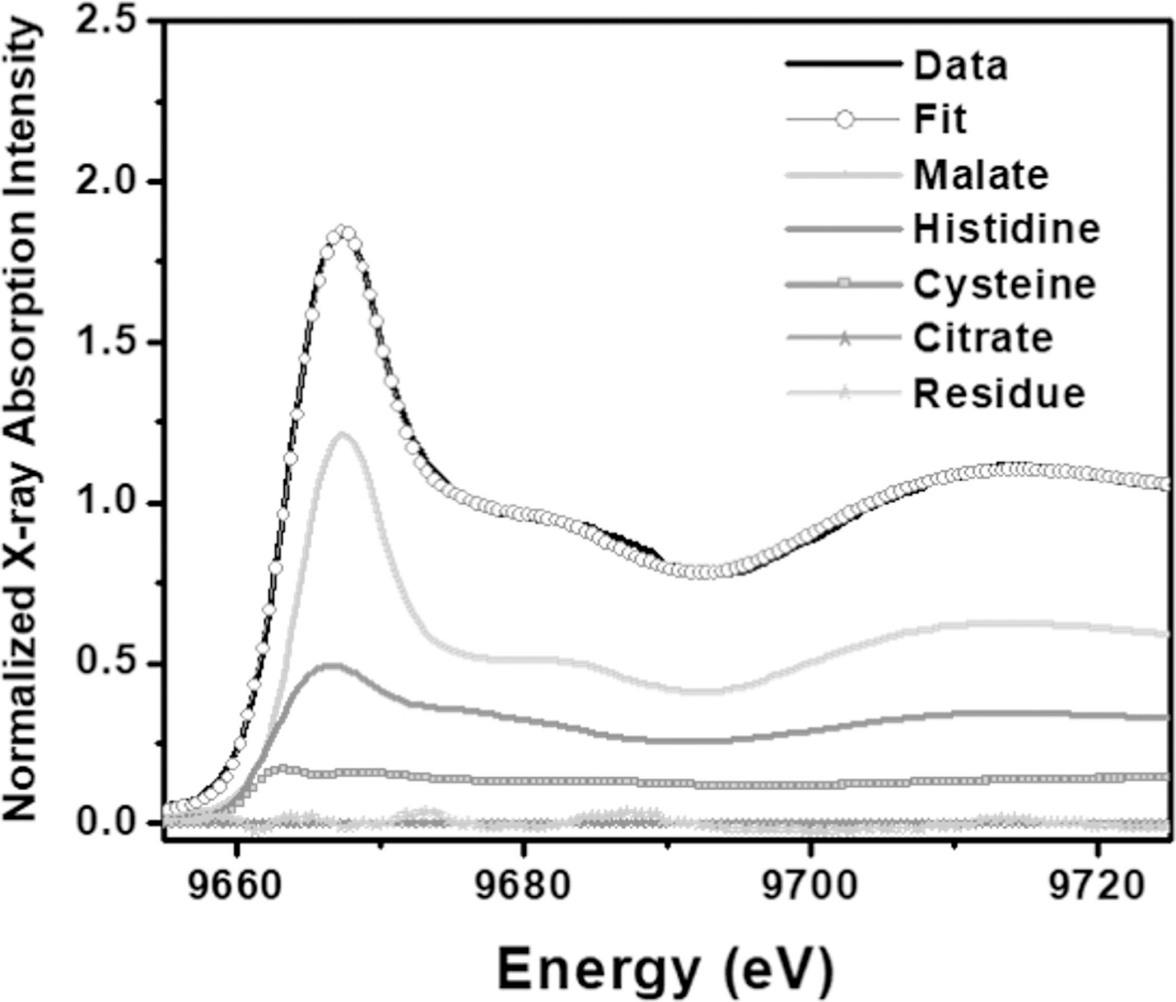
Linear combination fit of the spinach cotyledon XANES data with fit, components of fit, and residual.

Systematic differences were observed in the XANES spectra of spinach, clover, and radish cotyledons (Fig 4B). The edge energy position of radish (~ 9666.75 eV) cotyledon XANES was similar to the edge energy position of histidine (~ 9666.8 eV standard and the edge energy position of spinach (~ 9667.25 eV) was about half way between the edge energy positions of malate (~ 9667.8 eV) and histidine (~ 9666.8 eV) standards (Fig 4B, arrow 2). Additionally, a systematic decrease in the white line intensity concomitant with changes in the shape of the shoulder at 9675 eV from spinach to clover and radish data indicated that a significant fraction of the total Zn in cotyledons was likely complexed with histidine in radish and with malate in spinach (Fig 4B, arrow 2). Results of the linear combination fits (LCF) of XANES (Table 5) suggest that in the radish cotyledon, Zn-histidine complexes were approximately 4 times more abundant than Zn-malate complexes. In contrast, Zn-malate was almost twice more abundant than Zn-histidine in spinach and clover cotyledon. Inclusion of the Zn-cysteine standard resulted in significant improvement of the quality of the linear combination fit for all the three species. A representative LCF fit is shown for spinach in Fig 6. Zn-cysteine complexes accounted for 19% of total Zn in the spinach cotyledon and less than 10% in radish and clover. Most previous studies on Zn speciation in plants did not report complexation of Zn with cysteine in untreated plants [27, 29, 30].

**Table 5:** Linear combination fitting results for cotyledon.

Cotyledon	Malate	Histidine	Cysteine	Reduced Chi square (10^−3^)	R-factor
Spinach	54.3 ± 2.5%	27.0 ± 4.8%	18.7 ± 5.2%	1.6	0.1%
Clover	30.3 ± 1.7%	60.5 ± 3.6%	9.2 ± 3.0%	5.1	0.1%
Radish	16.9 ± 2.0%	78.9 ± 4.4%	4.9 ± 1.8%	7.5	0.1%

### Zn speciation of spinach and radish at different growth stages

Spinach and radish, where cotyledons were found to differ most in their complexation of Zn, were investigated further at four different stages of plant growth: dry seeds, sprouting seeds, leaves at 10 days and leaves at 90 days after sowing.

For radish, dry seed XANES data displayed remarkably different features compared to all standards examined in this study. Previous studies have suggested that many seeds contain Zn as a phytate complex [53]. The other three growth stages for radish resembled radish cotyledon (Fig 4C). Unlike radish seed, spinach dry seed XANES did not exhibit a sharp contrast with spinach XANES in different growth stages (Fig 4D). All the four growth stages for spinach resembled spinach cotyledon. Hence it is inferred that Zn is likely present as phytate complex in radish seed, and predominantly as a combination of malate and histidine complexes similar to those of radish and spinach cotyledons in other growth stages of the respective species.

#### Zn distribution in different plant parts

Since spinach leaf and radish root displayed highest bioaccumulation of Zn, they were chosen for μXRF imaging to understand the distribution and localization of the bioaccumulated Zn.

**Fig 7:**
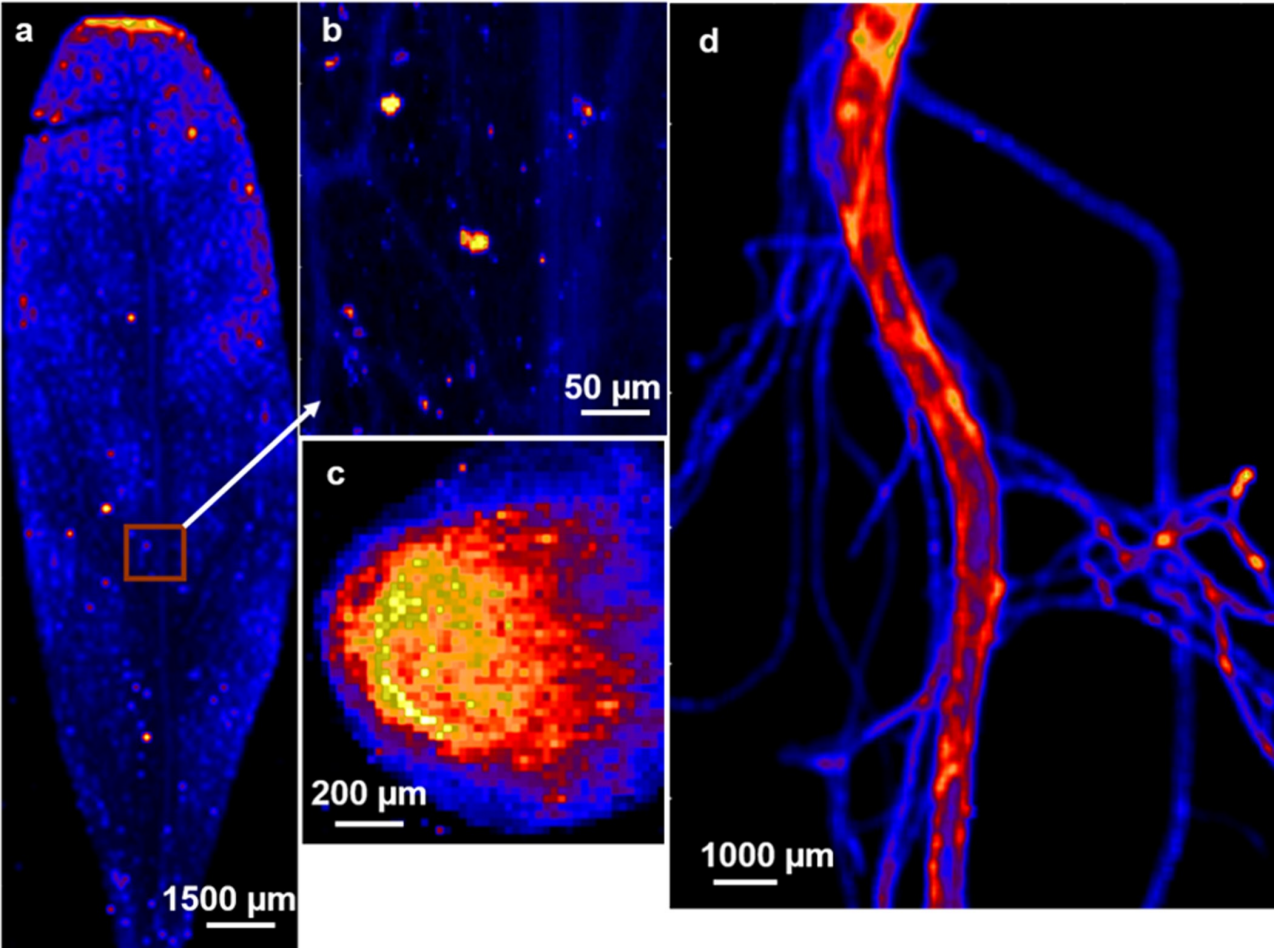
μXRF maps. (a) spinach leaf and (b) a high-resolution map of an area selected from spinach leaf (square area marked by arrow). μXRF map of radish root. (c) side view and (d) top view. Brighter areas indicate higher Zn concentrations.

In spinach leaves, μXRF maps show accumulation of Zn on the leaf tips (Fig 7A and 7B). Accumulation of Zn to toxic level in leaf tips are known to cause curling and/or decay of leaf tips [10], as seen in the spinach leaf. Additionally, a small portion of the spinach leaf near the main vein was mapped with 9 times higher (10*10 μm^2^) spatial resolution (Fig 7B). Several vacuoles of up to 200–400 μm^2^ are seen as local areas of high Zn loadings (‘hotspots’) in the leaf, in contrast with regions of lower Zn concentrations (non-hot spots) The compartmentalization of excess Zn in leaf vacuoles is a well-documented phenomenon ([25] and references therein). Difference between μXANES spectra of the hot spots and non-hot spots are not as dramatic as expected. It is, however, important to note that the μXANES spectra collected on the so called “non-hot spots” still have high enough loadings of Zn to facilitate good signal-to-noise ratio measured using a micron size x-ray beam, rendering them areas of moderate Zn loading. Significant differences in μXANES spectra of the hot spots compared to the bulk XANES data, which is an accurate representation of non-hot spots, (Fig 4E) illustrate changes in the speciation of Zn sequestered in vacuoles.

Comparison with standards suggest that Zn is sequestered primarily as a Zn-histidine complex within the vacuoles, while Zn-malate is the predominant species in the entire leaf. Difference between the average and local binding environments of Zn localized in vacuoles illustrates detoxification mechanisms employed by spinach leaf.

μXRF maps of a cross section of the main radish root (top view) and the finer radish hair roots (side view) are shown in Fig 7C and 7D. The top view of a cross section of the root shows that Zn did not accumulate in the root epidermis (Fig 7C), consistent with previously reported lack of accumulation of zinc in root epidermis for untreated plants [31]. The side view of the radish root showed that more Zn was present in the main root rather than in the root hairs or trichomes (Fig 7D). However, several areas of high Zn concentrations were found in the root hair. Considering that the thickness of main root was about two orders of magnitude greater than the root hair, hot spots on root hair had much higher Zn concentration per unit volume than the hot spots on the main root. Interestingly, μXANES spectra collected on the root hair are remarkably different from the ones collected on the main root (Fig 4F). Fig 5C and 5D show the data and fit of the FT magnitude and the expanded real part of the spectra collected on the main root and the root hair of the radish root. Compared to the FT of the main root, FT of the root hair is shifted towards longer radial distance (“to the right”), indicating a Zn-S coordination (Fig 5C). The radish root hair data was best fit with a mixed S (1.6 ± 0.4) and O/N (2.8 ± 0.5) coordination environment in the first shell (Table 6). A likely scenario is that on an average, about 40–50% of the Zn was complexed with cysteine or sulfide, while the rest of the Zn was complexed with either histidine or malate. The main root spectrum on the other hand exhibited tetrahedral coordination geometry with 4.3 (± 0.36) N (or O) atoms and 3.49 (± 0.86) C atoms at 3.02 (± 0.03) Å, suggesting Zn-histidine complexes (Table 6).

**Table 6:** μEXAFS fitting parameters for radish root.

Sample	Path	CN	R (Ǻ)	σ^2^(Ǻ^2^)	ΔE_0_ (eV)
Root Hair	N/O	2.76 (± 0.48)	2.03 (± 0.02)	0.0064 (± 0.0023)	2.3 (± 1.4)
	S	1.58 (± 0.42)	2.29 (± 0.02)	0.0082 (± 0.0030)	
Main Root	N/O	4.26 (± 0.36)	2.02 (± 0.02)	0.0062 (± 0.0023)	4.6 (± 0.87)
	C	3.49 (± 0.86)	3.02 (± 0.02)	0.0082 (± 0.0030)	

The root hair data presented in Fig 5C and 5D are an average of several (6–8) similar spectra collected on different locations of root hair. Sulfhydryl complexation of Zn in the radish root hair could suggest formation of Zn-sulfide nanoparticles in radish roots. If so, formation of Zn-sulfide nanoparticulate precipitates would be a likely detoxification mechanism for managing excessive Zn by radish roots. Although presence of Zn-sulfide nanoparticles, which could be only few nanometers in size, could not be directly confirmed due to the large spatial resolution of μXRF mapping (30 x 30 μm), high signal to noise ratio on radish root hair strongly suggest partitioning of Zn in solid phase as sulfide.

## Discussion

Accumulation of Zn, Cd, Cu, and Pb in different parts of edible plants grown in contaminated smelter soil has been studied for different species (spinach, radish and clover), and at different growth stages for radish and spinach. Zn K edge bulk XANES, μXANES, and μEXAFS, as well as μXRF maps were obtained to understand the speciation and distribution of Zn in edible plants.

The calculation of bioconcentration factors showed that while the concentrations in plants were lower than the total soil levels of Zn, Cu, Cd and Pb, they were significantly higher than the extracted soluble fraction in the cases of all metals except Cd. However, bioaccumulation in these edible plants was an order of magnitude less than the 1% criterion for qualifying as Zn hyperaccumulators [10]. Concentrations of Pb and Cd were also much higher than typically found in edible plants, suggesting that vegetables grown in contaminated soil would be dangerous for human consumption. Since smelter contaminated soil was used for this study, which was naturally co-contaminated with other metals, results presented in this study could be specific to soil co-contaminated with other metals (e.g., Cd). Specially, Zn and Cd have somewhat similar chemical properties, which could lead to inhibition of Cd uptake in presence of high level of Zn. However, it is also possible that certain species of plants might not have well developed mechanism to distinguish Zn and Cd, leading to higher uptake of Cd via zinc uptake mechanisms. Given the relative concentrations of Zn and Cd in the soils used for this study, it is remarkable that any Cd was taken up likely suggesting synergistic effect of Zn on Cd uptake. Interactions between co-contaminants in soil leading to synergistic and antagonistic metal uptake by plants is not well understood. It would make for an interesting additional study to decouple the effects of Cd stress on Zn uptake, distribution and localization in edible plants.

This study found a dynamic interplay between malate and histidine complexation for Zn in non-hyperaccumulating edible plants. Speciation of Zn varies significantly as a function of plant species. While Zn-histidine was four times more abundant than Zn-malate in spinach cotyledon, Zn-malate was twice about more abundant than Zn-histidine in radish cotyledons. Since, there is room for error in identifying Zn bound to O donor ligands in octahedral geometry as mentioned in published literature [34], we have collectively called Zn-malate complex as what could in principle be a combination of octahedrally coordinated O donors such as malate, citrate, and aqueous Zn. Nonetheless, signature for transition from complexation of Zn in octahedral (e.g., malate) to tetrahedral (e.g., histidine) geometry is rather precise.

Our results do not corroborate observations by Sarret et al., 2009 [34], where Zn was found to bind predominantly with O donor in octahedral geometry for all plant samples, as a combination of complexes formed with organic acids, cell walls or aqueous Zn. Another study by Terzano et al., [33] found that edible plants grown in contaminated soils contained Zn-phosphates and oxalates. Interestingly, speciation of Zn in plants grown on compost-amended changed to phytate, and citrate in roots, and as phosphate, cysteine and histidine in leaf cells. Taken together, these studies suggest that complexation of Zn in non-hyperaccumulating edible plants depends on the plant species as well as growth conditions including soil type and likely the chemical form of Zn in soil. Measurements conditions such as sample preparation could also lead to changes in the speciation of Zn. Many previous speciation studies were conducted under freeze dried or flash-frozen conditions [29, 33, 34]. Extensive sample preparation was avoided for this study to circumvent possible systematic errors. All samples presented in this study was conducted on fresh material under ambient conditions for improved accuracy in Zn speciation.

In agreement with previous findings, our results do not suggest significant changes in Zn speciation at different growth stages of plants [34]. Only notable observation as a function of growth stage was in radish seed, which likely changed from tetrahedral to octahedral coordination environment in other growth stages (sprouting seed, cotyledon, and leaf).

Perhaps one of the most important contribution of this study is direct elucidation of differences in speciation at regions of high Zn loadings compared to the rest of plant material. In spinach leaves, Zn-histidine was found to be the predominant local species at the hot-spots, as opposed to average coordination environment of Zn as Zn-malate. In spinach leaf, excess Zn seems to accumulate in vacuole and leaf tips rather than in the leaf veins (Fig 7A). In radish root, Zn is bound to malate in the main root and as cysteine or sulfide in the root hair which had much higher Zn loadings per unit volume (Fig 5C and 5D). Zn appears to have phase portioned to Zn-sulfide in the thin root hair likely as a detoxification mechanism to get rid of access zinc accumulation. However, it is possible that Zn was initially complexed with metallothionines or other protein residues, which eventually converted to Zn-sulfide NPs under prevailing biogeochemical conditions such as abundance of hydroxyl or catecholic groups. Regardless, complexation of Zn with histidine and cysteine in regions of higher loadings should not be surprising given their larger binding constants. Future studies should test whether strong complexing agents like histidine and cysteine are upregulated to bind excess zinc and compartmentalize in vacuoles or precipitate as sulfides in leaves and roots respectively.

Since plant derived biomass are ubiquitous in natural systems, these results indicate that the plant tissue-bound malate and histidine groups plays a key role in the speciation, fate, and bioavailability of Zn in aquatic and terrestrial ecosystems.

## Supporting information

S1 Toc (TIF)
