# Supplementary figures and images for: Uptake and speciation of zinc in edible plants grown in smelter contaminated soils
Source: PLoS One. 2020 Apr 17;15(4):e0226180. doi: 10.1371/journal.pone.0226180 (PMC7164604; doi:10.1371/journal.pone.0226180)

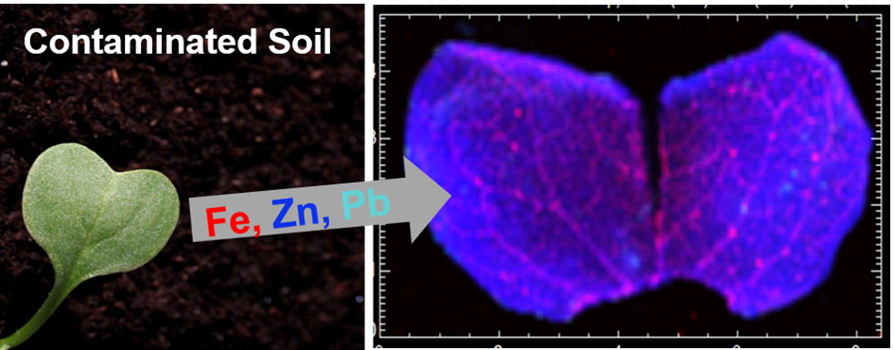

Supplement: S1 Toc — (TIF) [file pone.0226180.s001.tif]
